# A Policy Perspective on the Global Use of Smokeless Tobacco

**DOI:** 10.1007/s40429-017-0166-7

**Published:** 2017-08-31

**Authors:** Kamran Siddiqi, Aishwarya Lakshmi Vidyasagaran, Anne Readshaw, Ray Croucher

**Affiliations:** 1 Department of Health Sciences, The University of York, YO10 5DD, UK; 2 Barts and The London School of Medicine and Dentistry, Queen Mary University of London, London, UK

**Keywords:** Smokeless tobacco, Snus, Tobacco control, Global health

## Abstract

**Background:**

Globally, over 300 million people consume diverse smokeless tobacco (ST) products. They are addictive, cause cancer, increased cardiovascular mortality risks and poor pregnancy outcomes.

**Purpose of Review:**

To identify gaps in implementing key ST demand-reduction measures, focused literature reviews were conducted and findings synthesized according to relevant WHO Framework Convention on Tobacco Control (FCTC) Articles.

**Recent Findings:**

The literature supports implementation of ST demand-reduction measures. For taxation, labelling and packaging, most administrations have weaker policies for ST than cigarettes. Capacity to regulate ST contents and offer cessation support is lacking. There is poor compliance with bans on ST advertising, promotion and sponsorship.

**Summary:**

The literature on implementation of WHO FCTC for ST is limited. Although strengths of ST demand-control activities are currently identifiable from available literature, full implementation of FCTC is lacking. A wider evidence-based response to WHO FCTC is proposed, particularly for countries facing the greatest disease burdens.

## Introduction

“Smokeless tobacco” (ST) comprises tobacco products that do not involve a combustion process, such as chewing, nasal and oral tobacco [1•]. Globally, over 300 million people consume ST [1•], yet ST has been largely neglected in research and policy arenas because it is generally regarded as less harmful than cigarettes. It is also seen as a regional rather than a global problem, and too diverse and complex to control.

ST use is reported in at least 116 countries worldwide, including countries in Africa, Asia, Europe and the Americas [2•]. Smokeless tobacco consumption is therefore a global public health issue. In many countries in South Asia, ST is the most common form of tobacco used [3]. However, ST products vary widely in type and composition and there are marked regional differences in patterns of consumption. The different products have different addictive and carcinogenic properties. As well as nicotine, they may contain high quantities of carcinogens, notably tobacco-specific nitrosamines (TSNAs) [4] and heavy metals. pH levels vary between products, and pH is a key determinant of absorption of harmful chemicals from ST [4]. Other harmful ingredients are often included alongside tobacco, such as areca nut, which is a carcinogen in its own right. Consequently, there are substantial differences between the health risks of different ST products and their associated disease-burden across different countries and regions [2•]. For example, consumption of South Asian products (e.g. gutkha, zarda, paan, khaini) leads to a much greater health risk than in Sweden where ST use is equally prevalent, but the products (snus) contain much lower levels of TSNAs (see Table 1) [4].

**Table 1:** Common smokeless tobacco products

Gutkha – It is a preparation of crushed areca nut, tobacco, catechu, paraffin wax, slaked lime and sweet or savoury flavourings. Gutkha is consumed by placing a pinch of it between the gum and cheek and gently sucking and chewing.
Zarda – To manufacture zarda, tobacco leaves are shredded and boiled with lime and saffron. The mixture is then dried and mixed with finely chopped areca nuts. It is served in paan, which is chewed and spat out.
Paan – It is a preparation combining betel leaf with areca nut and sometimes also with tobacco. It is chewed and after chewing, it is either spat out or swallowed.
Khaini – A commercially manufactured product that contains shredded tobacco mixed with slaked lime, menthol and flavourings and sometimes with areca nut. The product is kept in the mouth for 10 to 15 min and sucked from time to time.
Snus – It is produced through a heat treatment process and contains tobacco, water, sodium carbonate, sodium chloride, moisturisers & flavourings. For consumption, it is placed between the gum and upper lip.

ST use leads to several different types of cancer [5]. An increased risk of cardiovascular deaths has also been reported [6]. ST use in pregnancy is associated with low-birth weight and stillbirths [7]. In 2010, ST use led to 1,711,539 disability adjusted life years (DALYs) lost and 62,283 deaths due to oral, pharyngeal and oesophageal cancers. Eighty-five per cent of these impacts occurred in South Asians [2•].

The World Health Organization (WHO) Framework Convention on Tobacco Control (FCTC) was enacted in 2005 [8]. This evidence-based international treaty obligates its signatories (currently 180 countries) to implement specific tobacco control measures. Countries implementing these measures to the highest level have seen a decline in smoking prevalence in recent years [9]. This cannot be argued for ST, because of several knowledge gaps [10]. Firstly, the evidence supporting WHO FCTC measures is mostly derived from research on smoked tobacco. Little is known about the transferability of these measures to ST. Secondly, while WHO reports on the extent to which tobacco control policies are implemented within member countries, there can be a lack of clarity as to whether these policies apply to ST as well as to cigarettes. Thirdly, where policies to control ST have been implemented, there is little published data on their impact.

This paper investigates the extent of these knowledge gaps for ST, in relation to the key demand-reduction measures outlined in WHO FCTC, and proposes a policy research agenda to control ST.

## Methods

We conducted a series of focused literature reviews, for articles published in the English language between 2012 and 2017, on key WHO FCTC demand-reduction measures for ST. PubMed databases were searched, along with reference lists of all included articles. Searches were conducted using the following keywords: (“smokeless tobacco” OR “chewing tobacco” OR “snus” OR “snuff”) combined with operationalised keywords for FCTC Articles 6, 9–14 (e.g. “tax,” “price,” “content,” “disclosure,” “packaging,” “labelling,” “warning,” “education,” “media,” “campaign,” “marketing,” “advertising” and “cessation”). Only studies reporting ST-specific outcomes were included, not dual use or use of ST for harm reduction, resolving any disputes about inclusion through discussion. A narrative synthesis of included studies was carried out according to relevant Articles (6, 9–14) of the Framework Convention. While assessed for potential biases, these studies were not given any formal quality assessment scores.

### Findings

We identified 1026 citations through database searching and screened 633 after removing duplicates. Another 467 citations were excluded after reviewing titles and abstracts. Key reasons for exclusion were papers based on product testing, harm reduction and dual use. We conducted full-text reviews on the remaining 166 papers and included 55 in our final narrative synthesis. Among these, 12 papers were relevant to FCTC Article 6 (price and taxation), 3 to Articles 9 and 10 (regulation of the contents of tobacco products and their disclosures), 8 to Article 11 (packaging and labelling of tobacco products), 4 to Article 12 (education, communication, training and public awareness), 21 to Article 13 (tobacco advertising, promotion and sponsorship) and 7 to Article 14 (demand-reduction measures concerning tobacco dependence and cessation). Most studies were either conducted in the USA, Nordic countries (Sweden, Norway) or in South Asia (India, Bangladesh and Pakistan). A range of research methods was used; however, most studies used cross-sectional designs.

#### Price and Taxation

Pricing and taxation measures (FCTC Article 6) are considered the most effective demand-reduction policy tools in tobacco control. This also applies to ST [11].

A US-based nationally representative survey of 14–18 year olds in 2012 highlighted that with every 10% rise in taxation, ST consumption fell by 12–18%, at least in the short term (consumption in the past 30 days) [12]. Referred to as price elasticity, this drop in consumption was greater for ST (− 1.2 to − 1.8) than for cigarettes (− 0.44 to − 0.60) [12]. Four studies from South Asia—one in youths and three in adults—supported the above findings. Based on the Global Youth Tobacco Survey, one study in India estimated that a 10% rise in the price of ST (gutkha) reduced its prevalence by 5.8% [13]. Another Indian study, based on the 2009 Global Adult Tobacco Survey, found that the total price elasticity of ST demand among adult men was − 0.212, slightly lower than that observed in youths [14]. The third study, based on the Consumer Expenditure Survey in India, found that leaf tobacco (surrogate for three ST products) showed the highest price elasticity in the poorest group (− 0.557). Hence, poor households were mostly likely to reduce ST consumption as a result of tax rises [15]. Similarly, the International Tobacco Control (ITC) study in Bangladesh indicated that a 10% rise in the price of ST (zarda) would reduce its use by 6% for cheap brands and by 4% for expensive brands [16].

Other studies have also shown similar results. A Swedish study, based on a mathematical “SimSmoke” model, estimated that a 70% rise in ST (snus) tax could reduce its use by 11.4% by 2040; the number of ST-attributable deaths averted between 2010 and 2040 could be up to 520 [17]. A US-based post hoc analysis of 2003–2009 National Consumer Survey data indicated that a tax rise could reduce ST consumption in adults (estimated elasticity of − 0.32) [18]. These results should be interpreted cautiously, as the study was primarily conducted to evaluate the effect of magazine advertising on ST use.

Where ST taxes have risen, ST sale and consumption have fallen. In Rajasthan, India, a small regional sample of ST vendors and consumers indicated a reduction in ST sales and consumption following a rise in ST price [19]. However, ST tax rises in India have been small, relative to the price increases of other commodities and per capita income, indicating a rise in general affordability [20]. A tax rise in Minnesota in 2013 resulted in price rises for ST equivalent to those for cigarettes, although tobacco companies used promotions to blunt the impact of taxation [21].

If tax increases are restricted to cigarettes only, there is a potential risk of substituting ST for cigarettes. The ITC study in Bangladesh indicated that a 10% rise in the price of cigarettes without a rise in ST (zarda) price led to a 3.5% higher ST (zarda) consumption [16]. Another study from India also showed that a rise in bidi (cigarette) prices alone will tempt customers to shift from bidi to ST use [15]. Similar concerns were raised in a US-based study, which reported a 17% increase in Google search queries for chewing tobacco, following the 2009 tax rise for cigarettes, which was far greater than that for ST [22].

Tax administration for ST is a particular challenge. In South Asia, ST is often manufactured in small and unlicensed units, with tax evasion and illicit trade being common. To overcome this challenge, the Indian government used a presumptive taxation approach, by introducing an excise levy for ST per manufacturing machine, which increased revenue collection fourfold [1•]. Other phenomena that could undermine the public health benefits of a tax rise on ST include unregulated internet sale of ST (for example, bypassing EU-laws [23]) and price promotions [21].

#### Regulation of the Contents of Tobacco Products and Their Disclosures

The literature reports challenges in regulating the contents of diverse ST products. Regulating all tobacco products for their contents and emissions through manufacturers’ disclosures and through regular testing and measurements are two key provisions of the WHO FCTC Articles 9 and 10. WHO FCTC expects its signatories to require ST manufacturers to disclose pH levels and toxicants (TSNAs, benzo[a]pyrene and nicotine), using recommended methods [24]. The WHO Study Group for Tobacco Product Regulations (TobReg) recommended upper limits for TSNAs and benzo[a]pyrene for all ST products [1•].

Currently, only nine WHO Member States regulate the content of ST products on the market [25]. For example, the USA requires all its ST manufacturers and importers to submit a list of all ingredients of their products. Under US laws, the Food and Drug Administration can establish limits on the amounts of nicotine, toxicants and other additives in ST products. Sweden has established its own voluntary manufacturing standards (for snus) called Gothiatek® [26]. These include maximum levels for certain undesired constituents; selection of approved raw materials, additives and flavourings; manufacturing standards to prevent contamination and public disclosure of all ingredients.

The South Asian and South East Asian regions where ST products are most popular have the most diverse ST market. However, they lack capacity to test and measure the ingredients of these products. A small step in this direction was made in 2011, when India invoked food safety laws to ban gutkha and tobacco-containing paan masala.

#### Packaging and Labelling of Tobacco Products

FCTC Article 11 ensures that the packaging and labelling of tobacco products provide accurate information about relevant constituents, display health warnings and do not mislead consumers about a product’s characteristics and ingredients. While considered to play a significant role in the appeal and risk perception of ST [27], the role of packaging and labelling has received little research attention so far. The study focus has been on health warnings, and we found three papers based on US-data and one from South Asia. One web-based survey of 1000 participants, stratified according to age group, found that health warnings and corporate branding had a significant effect on product appeal and people’s risk perception [28]. One of the US-based studies randomised participants into six groups and showed them graphic or text warning labels, “FDA approved” or “low-risk” endorsement labels, no labels or unrelated adverts on ST products [29]. Those in the graphic warning group showed the highest levels of perceived harm. The study recruited participants online and did not measure behaviour change. Based on a smaller sample of US male ST users, one randomised controlled trial (RCT) showed that those exposed to graphic health warning adverts were more likely to recall messages and less likely to have tobacco cravings, compared to those that saw text warnings only [30]. In a large study in India and Bangladesh, participants were randomised to receive either graphic pictorial warnings, symbolic pictorial warnings, text-only warnings or personal testimonial pictorial messages on ST products [31]. The participants perceived graphic pictorial warnings as the most effective. However, despite recruiting a large sample from one state, the study might not adequately represent the very large and diverse population of India.

Four policy evaluation studies on ST packaging and labelling were found. Two were based in the USA, where a text-only health warning appears on ST packs. The first one, based on the 2012 National Youth Tobacco Survey (NYTS), showed that only 40.3% of adolescents noticed any warning labels on ST products (“most of the time” or “always”). Among those who noticed, only a quarter thought about the serious health risks [32]. The effect was greater among adult ST consumers. Three quarters of those that participated in the 2012–2013 National Adult Tobacco Survey noticed the health warnings. Among them, three-quarters thought of health risks [33]. In India, graphic pictorial warnings replaced the symbolic scorpion warnings on ST products in 2011. However, these still only covered 40% of one side of the packaging and the images did not rotate frequently. Based on a cohort study, these changes were neither noticed nor considered provocative [34]. Conducted in four states on a large sample of 4733 ST users, the study findings are generalizable. A pilot survey among ST suppliers found that almost all ST health warnings in Bangladesh, Nepal and Pakistan were text-only; were often hidden, misleading, sometimes not in local languages; and did not comply with Article 11 specifications [10].

In implementing Article 11, most signatories have set a much lower requirement for ST than for cigarettes [1•]. A graphic pictorial warning, covering the majority and both sides of the packaging, and frequent rotation of images are not required.

#### Education, Communication, Training and Public Awareness

Education and raising awareness of the risks associated with consuming tobacco are important demand-reduction measures (FCTC Article 12). The “SimSmoke” model predicted that, combined with other policy measures, mass campaigning could reduce ST consumption significantly [17]. While some countries have launched ST awareness-raising campaigns, investment in mass media campaigns that could achieve high levels of awareness and bring a change in the social norms is lacking [1•]. We found two evaluations of mass campaigns against ST. Following a 6-week mass TV and radio campaign against ST in 2009 in India, a nationally representative large sample of ST users reported a significant impact. More than two thirds of ST users recalled the campaign adverts and almost three-quarters felt compelled to think about the health risks. Mass campaigns through digital media can be run at low cost and offer greater reach within a younger audience. A process evaluation of an online media campaign (ChewOnThis.in) showed that the campaign website had 10,949 visits and 1131 registrants in 6 weeks [35].

FCTC Article 12 requires governments to ensure that health professionals also receive sufficient education about ST and its associated harms. However, such awareness among medical professionals is deficient and almost a quarter of physicians in India may advise smokers to switch from cigarettes to ST as a “cessation aid” [36].

#### Tobacco Advertising, Promotion and Sponsorship

The key measure in FCTC Article 13 is a comprehensive ban on tobacco advertising, promotion and sponsorship (TAPS). The ban covers point of sale displays, the Internet, brand stretching, product placement, corporate social responsibility, and tobacco in entertainment media. Despite strict regulations, the tobacco industry continues to invest heavily in marketing its products, including ST. A sum of $20 million was spent on advertising ST in the USA between June–September 2012, mostly targeting middle-aged white men, with a strong theme of masculinity [37]. An analysis of over 350 magazines printed between 2005 and 2009 found 861 adverts for ST (snuff), with more adverts for discount products targeted at youths [38]. Web traffic on sites promoting ST (snuff) rose between 2011 and 2014 [39]. ST was also promoted in online video/banner tobacco adverts [40] and in YouTube videos [41, 42]. Advertising ST over the Internet, in print media and at the points of sale enhances ST curiosity [43], experimentation [44] and uptake among youths [45, 46]. In late adolescence, exposure to ST adverts is associated with ever ST use, and in young adults, it is associated with regular ST use [47]. Among general adults, such exposure increases ST consumption [14, 18, 48].

We found nine studies that assessed compliance with national policies on TAPS. The methods included consumer surveys and ST supplier assessment. When asked about exposure to any ST advertisement in the previous 6 months in a large survey in two states in India, 74% of adults responded affirmatively [49]. Two surveys focussed on schools; one found that 41% of shops/stalls within 100 m of schools in India sold ST and out of these, more than half advertised ST at point of sale [50]. The other survey found that 54% of schools had at least one ST advert within 100 m [51]. Self-reported ST consumption among children was associated with the density of these adverts.

Of four US-based studies, three assessed compliance by observing the proportion of outlets that advertised ST at point of sale and one did so before and after the relevant FDA regulations came into place. The first study found that 57% of 129 tobacco outlets in Ohio displayed ST advertisements at point of sale [52]. The next two studies found point of sale advertising for ST in 21 and 30% of tobacco outlets, respectively [53, 54]. The fourth study observed a marked reduction in the number of advertisements at point of sale within tobacco outlets in Ohio, following the introduction of the Tobacco Control Act 2009 [55].

In Norway, 98% of outlets were compliant after the introduction of a point of sale display ban on ST (snus) [56]. In a pilot study comprising structured interviews with ST suppliers in Bangladesh, Nepal and Pakistan, product display was found to be the main form of point of sale advertisement. Also, ST manufacturers offered promotions (bulk-buy discounts and prize draws) to the vendors [57]. The above surveys were limited in their generalizability and since most were conducted at one time-point, any associations cannot be extrapolated as causal.

#### Demand-Reduction Measures Concerning Tobacco Dependence and Cessation

FCTC Article 14 requires governments to promote tobacco cessation and provide adequate support to those who are dependent on tobacco and wish to quit. However, cessation support remains the most poorly implemented measure worldwide [58]. Fewer than 50% of signatories to the WHO FCTC have implemented Article 14 [58]. Poorer countries have far less cessation support than high-income countries.

Most ST products contain a high nicotine concentration [4] and regular users show strong dependency [59, 60]. Compared to smoking cessation, the evidence for ST cessation is limited and complicated by ST heterogeneity [61]. A Cochrane review [62], while concluding that pharmacotherapies and behavioural support may help ST users to quit, did not include any South Asian studies. The authors recommended further research on different types of ST. ST products in South Asia are highly alkaline, enhancing nicotine absorption and making them more addictive than products used elsewhere [59]. Moreover, there is a strong socio-cultural dimension to ST use in South Asian populations. Both factors may feature in the effectiveness of any cessation support offered, but have rarely been considered.

Two UK non-RCTs found that behavioural support and nicotine replacement therapy (NRT) were non-significantly more effective than brief advice [63], and NRT predicted short-term abstinence [64]. The latter findings were replicated in a multi-centre cohort study across South Asia [65]. A feasibility study found a culturally appropriate behaviour change intervention was acceptable to ST users in Pakistan and the UK [61]. One Indian RCT observed no difference in quit rates at 12 weeks (varenicline vs. placebo) [66], and a second showed a small effect of a community outreach intervention on abstinence at 6 months [67]. In the absence of RCT evidence, the UK National Institute for Health and Care Excellence (NICE) guidance [68] for ST cessation recommends behavioural support only and excludes the use of pharmacotherapies, but has recommended research to assess these.

## Discussion

The primary aim of this narrative review was to identify the published literature relating to the use of WHO FCTC demand-reduction measures to control ST. It reports the first overview of this issue. Although not a systematic review, clear and pre-defined methods were followed in the conduct of this research. Grey literature and papers not published in the English language were omitted.

The research has established the following key findings. Firstly, the literature is limited in content, with only 55 papers identified. Most of the identified papers reported cross-sectional study designs, with their recognised potential for recruitment selection and recall biases and an inability to establish causality. Secondly, the literature reports research primarily conducted outside South Asia, the geographic region with the highest ST prevalence and subsequent greatest burdens on health. Thirdly, most of the papers identified focused on WHO FCTC Article 6 (pricing and taxation) and Article 13 (TAPS). In contrast, WHO FCTC Article 14 (tobacco cessation) has been substantially overlooked with respect to ST.

Recognising these limitations, the following substantive findings can be cautiously proposed. Increasing ST taxation and price will reduce demand. The price elasticity of ST is reported to be greater than smoked tobacco. Graphic pictorial warnings on packaging, compared to other forms of health warnings, are more effective. Implementing point of sale regulations reduces exposure to ST advertising.

These findings are important because they demonstrate the possibilities for ST demand-reduction measures. In the last 10 years, WHO FCTC measures have contributed to a substantial reduction in smoking prevalence in many countries [9]. In contrast, most countries are observed to have either not implemented these measures for ST, or have set standards much lower than those for cigarettes. ST taxes have been raised, although not as much as cigarette taxes, while health warning requirements are less strict. Other international and regional reports have similarly identified the challenges in regulating ST products [69•, 70].

Areas for a ST demand-reduction policy implementation and research agenda can be identified for future action. Ensuring and evaluating compliance with the current WHO FCTC Articles should be rigorous. The findings identified from this review should be further developed, adopting more rigorous study designs, where appropriate. In addition, building laboratory capacity to enable further ingredient analysis of the diverse range of ST products should be undertaken. In turn, accurate and unambiguous ingredient analyses can inform the robust development of warning labels and point of sale regulation.

In conclusion, this narrative review has identified a limited body of the literature dealing with the implementation of the WHO FCTC with respect to ST. While most of the WHO FCTC demand-reduction measures are applicable to ST, their implementation is lacking compared to that for cigarettes. The findings demonstrate some current strengths of ST demand-control activity. A need to build upon these current strengths and also develop a wider evidence-based response to WHO FCTC is proposed, particularly for countries facing the greatest disease burdens.
